# Completing the Circle of Industrial Service

**Published:** 1921-07

**Authors:** A. W. Shraep

**Affiliations:** Director of Research Division, Industrial Dental Clinics, The Sam’l A. Crocker Co.


(This is the first of a series of three articles.)

### COMPLETING THE CIRCLE OF INDUSTRIAL SERVICE

BY A. W. SHRAEP

Director of Research Division, Industrial Dental Clinics,
The Sam'l A. Crocker Co.

It is comparatively not so very long ago that there was started among their employees by the heads of a few industries on a limited scale what has been called ''welfare work.'' Permit me to hereafter call this work ''Industrial Service,'' since that is what it actually amounts to, viz: ''service''—and since this article concerns itself with service rendered to workers in the industrial field, it should be spoken of as ''industrial service'' and in every case this expression should take the place of so-called ''welfare work'' which very frequently has not been cheerfully received because of the slight odium of charity attached to it.

Slowly but firmly has the idea of ''industrial service'' found its place in the industries of our country and from time to time has been extended and broadened in its scope because executives gradually began to realize that all the different phases which to-day are embraced by ''industrial service'' make for better, healthier, more efficient and loyal employees.

Medical science has for many years played its part in ''industrial service'' by either qualifying or rejecting the prospective employee, in some cases by means of a rather superficial examination. In later years, however, it has come into its own by not merely admitting healthy prospects but—and this work is of great importance—by helping temporarily unfit applicants to get onto their physical feet, and by doing its best to keep them there.

And now permit me to digress for a moment. Let me ask you to pick up any manufactured article nearest your hand. Upon being asked as to what enters into the finished article, your reply will probably be the same as the answer I have received from every single individual to whom I have personally put the same question, viz.: ''Raw material and machinery.'' True, raw material and machinery are important elements which go into the making of your finished article, and in order to secure the very best of each, many an executive maintains an elaborate research department, knowing that without such a department probable and possible success may be turned into certain failure.

Since the writer is neither a member of the medical nor of the dental profession (with whose work this series of articles is principally concerned) but one to whom efficiency, either individual or collective, is that which makes the world a place worth working in, permit me to correct this almost usual analysis of a finished product. ''It is the 'human element' co-operating with the 'mechanical element' upon a 'suitable raw product' which produces the finished article.'' The manufacturer through his research department assures himself beyond a question of doubt that he has the right and most efficient mechanical element and the most suitable raw product at the lowest price possible. But how about the *initial* and *sustained* efficiency of the ''human element?'' The least amount of consideration is usually given the ''human element'' which in my opinion really is the nub of industrial life, not merely because without this the other two are useless, but because the maintenance of the human element at its highest point of efficiency will bring the highest possible returns on the investment in the other two elements.

And where to-day experimental departments, investigation and location of new and possibly better and more convenient sources of raw materials have reached their highest level, because expense or capital investment in this field did not count—installation of ''industrial service'' departments have, with few exceptions, been sadly neglected.

No matter in how many other ways the "industrial service" looks after the employees, obtains closer co-operation with the employing company, keeps them good-natured and increases their sense of loyalty and endeavors to increase their efficiency, it is the medical department which plays the most important role in "industrial service."

Some medical departments are used merely to separate the wheat from the chaff, the healthy chap being accepted while the unfortunate one, weakened by *curable* illness or through *undernourishment* is advised to hunt another job. In other concerns the medical department goes a step further and orders this type of man placed at some light occupation, gives him treatment, instructs him how to take care of himself, thus saving another member of society and by such methods building up a nucleus for a loyal force and lowering the turn-over. Of course, a department of this character handles all emergency cases on the ground, whether caused by accident or sudden illness.

In medical departments of still another character the service is carried into the home of the employee.

And it is the executive of to-day who first considered "industrial service" a sort of "foolish idea" or the use of it as "catering too much to the working class"—it is this man who has discovered that "it pays."

There now arises the question: "Is a medical department of the best character under competent leadership and used to its fullest extent, complete?" Sore feet and sore fingers, sore heads and sore backs and all manner of internal and external complaints receive due attention—*with the exception of the mouth!*

Of the two thousand or more concerns in the United States which maintain through the "industrial service" a medical or hospital department *only* 156 have installed a dental clinic as an absolutely necessary part of the medical service and there are very few medical directors indeed, who to-day could or would do without it, and it is the sincere opinion of the writer that ''industrial service'' operating a medical department without a dental clinic is far, very far from complete, in fact, a dental clinic is of such importance that one medical director under whose supervision the entire ''industrial service'' is operating allows the dental clinic a valuation of *twenty-five per cent.* out of a total of one hundred per cent. for the *entire* service.

It is to-day a proven fact that many diseases, some of them until very recently called or considered ''chronic or incurable,'' are in a large percentage attributable to decayed teeth or some other unsound condition of the oral cavity and have yielded under dental treatment, where purely medical treatment has been able to give only relief. Just to mention a few of the diseases with which the average man is familiar, there are among them rheumatism, chronic indigestion, heart disease, kidney troubles, hardening of the arteries, yes, and even insanity.

If our people have not yet come to a full realization of the facts involved, they have at least recognized the danger ahead of them and that measurements must be taken not merely to effect a cure but to prevent the disease which is entirely within the province of possibility. ''Oral Hygiene'' is to-day being carried on in our schools all over the country, in institutions of various characters and even in penal institutions. And this work should be and *must* be, as part of the medical departments, as part of the true ''industrial service,'' extended to the man and woman in the factory.

Every industry wants the most efficient mechanical element possible to accurately perform certain operations on a raw product, insists that such mechanical element be at all times maintained at its highest point of efficiency.

And again the plain question, what about the ''human element with its *native* or *acquired* efficiency?''

In the next article of this series the writer shall show examples from different parts of the country where the circle of "industrial service" has been completed through the addition of a dental clinic to the medical department, of work done in these clinics and cures of physical ills accomplished, taken from the records of various industries and also from the army and navy, all concrete facts which should, if this article be not sufficient to cause some awakening somewhere, be of help to "industrial services" in discussing with the management of their institutions the proposition of "Completing the Circle."

And to the executive, reading this article, I submit these questions: "Why did you first go to your dentist? Why did you follow up your first visit with others for prophylactic treatment and inspection?"

"Did that seeming mental fatigue, that only too apparent physical laziness disappear, and did not that old time vigorousness with its joy of life and the old pep come back better than ever?"

What does either condition in *your* employee mean to *you* in dollars and cents?

Correspondence regarding this article or the subject matter will be handled promptly.

---

To REFINE WAX. I was very much surprised several days ago to note one of my friends, a dental practitioner, placing some used wax in the discard. After questioning what appeared to me as a very extravagant habit, it developed that this waste was nothing uncommon among our contemporaries. For several years I have been accustomed to dropping about five normal sized drops of sulphuric acid into the wax-pot preparatory to boiling and making sheets out of the wax, which I have found to be as fit for use as the wax one can buy as new.—Dr. J. J. Fischman, in *Digest*.

